# National "Unfits"

**Published:** 1920-12-18

**Authors:** 


### National "Unfits."

### THE PRIME MINISTER'S COMPARISONS.

The medical correspondent of the *Times* challenges the statement made in the House of Commons the other day by the Prime Minister that we had "the largest percentage of unfits of any population in Europe." Now this statement was no doubt based on the statistics furnished in the last few months of the war by the Ministry of National Service, and the only figures available are those covering the period November 1917 to October 1918. The *Times* correspondent is, therefore, right in pointing out that this is an inadequate basis for so sweeping a statement, and that it is in no way possible to institute an exact comparison between the physique of this country and that of other countries engaged in the Great War, because the methods of testing individuals varied in strictness and conditions at different times in different countries.

At the same time, there is no reason to become hysterical on the subject and to suggest that the Prime Minister was giving "a view of England which is insulting to our pride." The fact of the matter is that nothing which he might say could be more painful to our national *amour propre* than those same figures which were published by the Government, and analysed with cold severity by, among others, Professor Keith. They do not positively show that the physical condition of the British population is materially worse than that of other nations, but they do show that the standard is alarmingly low almost throughout industrial Britain; and, at the time, they came upon the comfortable complacency of our people with the shock of a thunder-clap. The argument that all the best physical types went to the war in the early stages,

**THE PUBLIC WELL-BEING**—(*continued*).

and that therefore the later examinations do not form anything like a true criterion, is an assumption not easy to justify. The present writer knows from a long personal experience of the Army, dating from the beginning of hostilities, that a very large proportion of the men who joined up at the outset in a spirit of generous enthusiasm appeared utterly unfit for service, not only because they were too old but by reason of debility or definite physical defects. But the sieve was very large, and they managed to pass through it. They went to France and Belgium; and a great many of them, owing to the peculiar conditions of the campaign, and the magnificent organisation of the medical service, not only did splendid service as soldiers, but maintained their military efficiency long after it seemed possible that they could endure the hardships of the trenches. On the other hand, a large number failed to endure them long; and many of these, and others who were weeded out, were transferred to administrative corps behind the lines, used in a light capacity, or sent home. The division in which the writer served contained, as early as 1915, a surprising number of enthusiastic unfits, and most of them soon became a difficult problem and a drag on efficiency.

Therefore, it seems to be going further than is legitimate to assume, merely because more intensive methods of medical examination were employed towards the close of the war, when conscription was in general operation, that the classification revealed in this period does not in any way represent the average conditions of things. What it chiefly served to show was the bad effect which modern industrial life produces on industrial populations in this country. We shall not know, until or unless periodical medical examinations for the whole population are made in this country and abroad on an equal basis, how Great Britain compares with its late enemies or its allies or neutrals in the matter of physique. But the rest of the civilised world must be in a pretty bad way if—as seems to be suggested without data by the Prime Minister's critic—Great Britain in its national physique "compares favourably" with other nations. The probabilities are, indeed, in the opposite direction, for Great Britain is a small, densely populated, and essentially industrial country, tending to become still more industrialised in the process of time. And it is precisely these industrial conditions which have bred overcrowding, disease, low vitality, and physical degeneration. It may be unnecessary, but it is difficult to exaggerate the situation; and if, instead of crying out about insulting the British race, we face the disagreeable facts and try to analyse the causes, we shall be able to focus public attention on the matter and do something to counteract the evils of super-industrialism. The chief danger is not that our standards should be over-emphasised. The danger is lest they should continue to be underestimated. The need is urgent for a few persons with the power of Mr. Lloyd George, no matter what their politics, to sound the warning bell.

